# Draft Genome Sequence of a *Bacillus* Bacterium from the Atacama Desert Wetlands Metagenome

**DOI:** 10.1128/genomeA.00955-15

**Published:** 2015-08-20

**Authors:** Claudia Vilo, Alexandra Galetovic, Jorge E. Araya, Benito Gómez-Silva, Qunfeng Dong

**Affiliations:** a Agriaquaculture Nutritional Genomic Center (CGNA), Genomics and Bioinformatics Unit, Temuco, Chile; b Department of Biological Sciences, University of North Texas, Denton, Texas, USA; c Laboratorio de Bioquímica, Departamento Biomédico, Facultad Ciencias de la Salud, Universidad de Antofagasta, Antofagasta, Chile; d Center for Biotechnology and Bioengineering (CeBiB), Universidad de Antofagasta, Antofagasta, Chile; e Departamento Tecnología Médica, Facultad Ciencias de la Salud, Laboratorio de Parasitología, Universidad de Antofagasta, Antofagasta, Chile; f Center for Biomedical Informatics, Department of Public Health Sciences, Stritch School of Medicine, Loyola University of Chicago, Maywood, Illinois, USA

## Abstract

We report here the draft genome sequence of a *Bacillus* bacterium isolated from the microflora of *Nostoc* colonies grown at the Andean wetlands in northern Chile. We consider this genome sequence to be a molecular tool for exploring microbial relationships and adaptation strategies to the prevailing extreme conditions at the Atacama Desert.

## GENOME ANNOUNCEMENT

Colonies of a *Nostoc* cyanobacterium grow naturally at wetlands >3,000 m of altitude at the Andes Mountains range in South America. These colonies are harvested, sun dried, and sold as Llayta for human consumption. This culinary practice from Andean communities in northern Chile and southern Peru can be traced back to pre-Columbian times (1, 2).

We isolated a *Bacillus* bacterium from the complex microbiota associated with Llayta colonies and decided to sequence its genome in order to have a molecular tool to address physiological relationships between the dominant Llayta cyanobacterium and its associated microbiome. Also, it will allow the exploration of microbial survival strategies to extreme dryness, arsenic, heavy metals, UV radiation, and other prevailing extreme environmental conditions in the Atacama Desert (3).

We sequenced the *Bacillus* species genome via MiSeq sequencing technology using paired-end libraries, with an average insert size of 250 bp. The sequencing produced 16,279,922 reads with a total length of 4,883,976,600 bp. Based on the estimated genome size of members of the *Bacillus* genus, the sequencing achieved about 600-fold coverage of the genome. Sequencing was done at the Greehey Children’s Cancer Research Institute (GCCRI) next-generation sequencing (NGS) facility at the UT Health Science Center at San Antonio, TX.

The sequencing reads have an average length of 300 bp, with good quality scores, as evaluated by the FastQC program (version 0.10.0 [http://www.bioinformatics.babraham.ac.uk/projects/fastqc/]). Assembly of the reads was performed with the A5 software, which was designed to use with sequencing reads produced by illumina sequencing technology (4). The assembler includes a pipeline that automatically performs data cleaning, error correction, assembly, and quality control (4). The longest scaffold obtained was 1,286,564 bp long, with an average scaffold length of 19,881 bp. The final 290 scaffolds assembled were annotated using the Rapid Annotations using Subsystems Technology (RAST) server version 4.0 (5).

The assembled draft genome is 5,802,366 bp long. Its G+C content is 41.2%, and the genome contains 5,945 protein-coding genes and 92 tRNA genes. Analysis of the 16S rRNA gene showed that this *Bacillus* sp. from the Atacama wetlands is closest to *Bacillus halodurans* and *Bacillus selenitireducens* in the phylogenetic tree. The metabolic capabilities that are in dominant proportions include the metabolism of amino acids (14%), carbohydrates (12%), proteins (10%), and RNA (7%). We observed genes related to dormancy and sporulation capacity, which might confer resistance to the extreme dry conditions of the Atacama Desert (6).

Interestingly, we observed genes related to arsenic resistance, which indicate the capability of the bacterium to survive in the heavily contaminated Atacama Desert. Some of the genes include those for the arsenate reductase, arsenic efflux pump protein, arsenical pump-driving ATPase, arsenical resistance operon repressor, arsenical resistance operon *trans*-acting repressor ArsD, and transcriptional regulator from the ArsR family. In addition, genes related to copper and chromate transportation were observed, which indicate additional metal resistance capabilities of the *Bacillus* species (7).

### Nucleotide sequence accession numbers.

The draft genome sequence of the *Bacillus* sp. has been deposited at DDBJ/EMBL/GenBank under the accession no. LFEL00000000. The version described in this paper is the first version, LFEL01000000.
